# AMPK and Beyond: The Signaling Network Controlling RabGAPs and Contraction-Mediated Glucose Uptake in Skeletal Muscle

**DOI:** 10.3390/ijms25031910

**Published:** 2024-02-05

**Authors:** Leon Peifer-Weiß, Hadi Al-Hasani, Alexandra Chadt

**Affiliations:** 1 Institute for Clinical Biochemistry and Pathobiochemistry, German Diabetes Center (DDZ), Leibniz Center for Diabetes Research at Heinrich Heine University, Medical Faculty, 40225 Düsseldorf, Germany; leon.peifer-weiss@ddz.de (L.P.-W.); hadi.al-hasani@ddz.de (H.A.-H.); 2 German Center for Diabetes Research (DZD e.V.), Partner Düsseldorf, 85764 Neuherberg, Germany

**Keywords:** AMPK, RabGAPs, contraction, glucose uptake, skeletal muscle

## Abstract

Impaired skeletal muscle glucose uptake is a key feature in the development of insulin resistance and type 2 diabetes. Skeletal muscle glucose uptake can be enhanced by a variety of different stimuli, including insulin and contraction as the most prominent. In contrast to the clearance of glucose from the bloodstream in response to insulin stimulation, exercise-induced glucose uptake into skeletal muscle is unaffected during the progression of insulin resistance, placing physical activity at the center of prevention and treatment of metabolic diseases. The two Rab GTPase-activating proteins (RabGAPs), TBC1D1 and TBC1D4, represent critical nodes at the convergence of insulin- and exercise-stimulated signaling pathways, as phosphorylation of the two closely related signaling factors leads to enhanced translocation of glucose transporter 4 (GLUT4) to the plasma membrane, resulting in increased cellular glucose uptake. However, the full network of intracellular signaling pathways that control exercise-induced glucose uptake and that overlap with the insulin-stimulated pathway upstream of the RabGAPs is not fully understood. In this review, we discuss the current state of knowledge on exercise- and insulin-regulated kinases as well as hypoxia as stimulus that may be involved in the regulation of skeletal muscle glucose uptake.

## 1. Exercise-Regulated Glucose Uptake in Skeletal Muscle Is Crucial for Controlling Systemic Glucose Levels

Maintaining stable blood glucose levels is important to ensure physiological body function and sufficient energy supply to meet changes in energy needs. Therefore, hormonal and metabolic signals regulate the mechanisms that raise and lower blood glucose. One regulatory mechanism is the secretion of insulin by the islets of Langerhans, which is stimulated by a postprandial rise in blood glucose levels and induces elevated blood glucose uptake in peripheral tissues (reviewed in [1]). Skeletal muscle is responsible for the vast majority of insulin-stimulated glucose disposal, but it is also unique in that it exhibits increased glucose uptake in response to exercise and muscle contraction [2,3]. Insulin resistance (IR), a condition in which peripheral tissues lose their ability to absorb glucose from the bloodstream in response to insulin stimulation, plays a crucial role in the development and progression of metabolic diseases such as type 2 diabetes. IR can occur undetected in pre-diabetics years before the disease develops [4,5,6]. Chronic hyperglycemia caused by insulin resistance leads to serious complications such as retinopathy, cancer, cardiovascular disease, neuropathy and nephropathy (reviewed in [7,8,9,10,11,12,13,14]). Interestingly, exercise-stimulated glucose uptake in skeletal muscle is not impaired in insulin-resistant subjects [15] (reviewed in [16]). Exercise is therefore an excellent therapeutic option for both diabetic and prediabetic individuals [17]. The intracellular pathways by which insulin stimulation and exercise induce glucose uptake in skeletal muscle are distinct but appear to converge at TBC1D1 and TBC1D4 (also known as AS160, AKT substrate of 160 kDa), two key signaling molecules critical for the regulated trafficking of the facilitative glucose transporter type 4 (GLUT4) [18,19,20]. Phosphorylation of the two Rab GTPase-activating proteins (RabGAPs) by insulin- and contraction-sensitive kinases leads to increased translocation of GLUT4 to the plasma membrane and consequently to increased uptake of glucose into the cell (reviewed in [21]). To date, at least a dozen phosphorylation sites have been identified in TBC1D1 and TBC1D4, most of which have been described as targets for the serine/threonine kinases AKT and the AMP-dependent kinase AMPK (reviewed in [22]). The regulatory mechanisms underlying the phosphorylation of RabGAPs that mediate exercise and insulin signaling are largely unknown. Only a few serine and threonine sites in TBC1D1 and TBC1D4 have a specific upstream kinase described.

## 2. Insulin and Contraction Signaling Converge at the Regulation of GLUT4-Mediated Glucose Transport

GLUT4 is responsible for the regulated glucose uptake into insulin-responsive peripheral tissues including adipose tissue and skeletal muscle, as well as the heart [23]. In the unstimulated, basal state, GLUT4 is retained in the cytoplasm, where it is stored in intracellular storage vesicles (GSVs). Various stimuli can induce GSV translocation to the plasma membrane, leading to GLUT4 incorporation into the membrane, thereby enhancing glucose uptake [24,25]. In skeletal muscle, known stimuli include muscle contraction, calcium-related mechanisms and insulin signaling. The latter activates the insulin receptor, leading to phosphorylation of insulin receptor substrates (IRS) and subsequent activation of phosphatidylinositol 3-kinase (PI3K). PI3K initiates the generation of phosphatidylinositol-3,4,5-triphosphate, which recruits phosphoinositide-dependent kinase-1 (PDK1) to activate the serine/threonine kinase AKT (reviewed in [26]). Upon activation, AKT phosphorylates a variety of downstream targets, including the RabGAPs TBC1D1 and TBC1D4 [18,20,27]. The main role of RabGAPs is to catalyze the intrinsic GTPase activity of small RabGTPases, which are versatile regulators of cellular vesicle trafficking (reviewed in [28]). In the basal, unphosphorylated state, TBC1D1 and TBC1D4 render GSV-associated RabGTPases in their inactive, GDP-bound state, thereby inhibiting GLUT4 translocation [29]. Of the more than 60 Rab GTPases described to date (reviewed in [28,30]), a subset has been shown to be associated with GSVs, namely Rabs 2A, 8A, 10, 11, 13, 14 and 28 [31,32,33,34]. Phosphorylation of RabGAPs at multiple serine (Ser) and threonine (Thr) sites leads to dissociation of RabGAPs from GSVs [32,35]. The resulting accumulation of downstream RabGTPases in their active GTP-bound form leads to the induction of GLUT4 translocation to the plasma membrane and, as a consequence, to increased cellular glucose uptake (reviewed in [36]). To date, at least 15 phosphorylation sites have been experimentally identified for TBC1D1 and TBC1D4 (reviewed in [22]). As major convergence points of insulin and contraction signaling, most of the phosphorylation sites identified so far have been attributed to either AKT or AMP-activated kinase (AMPK) as upstream effectors [18,19]. An overview of the known phosphorylation sites has previously been reviewed by Espelage et al. [22]. 

## 3. Kinases Regulating RabGAPs and Contraction-Stimulated Glucose Uptake

### 3.1. AMPK Is Sufficient but Not Necessary for the Regulation of TBC1D1 and TBC1D4

AKT and AMPK are activated by different stimuli and pathways. AKT is part of the insulin signaling pathway, while AMPK has been shown to phosphorylate TBC1D1 and TBC1D4 in response to exercise to adapt to increased energy demands by enhancing GLUT4-mediated glucose transport [20,37]. One effect of exercise is to increase energy expenditure in skeletal muscle, leading to an elevated ratio of AMP/- to ADP/ATP, which supports AMPK phosphorylation at Thr172 by liver kinase B1 (LKB1) [38,39,40], thereby promoting its kinase activity through allosteric activation (reviewed in [41]). The involvement of LKB1 in AMPK activation was initially demonstrated in mice with a muscle-specific LKB1 knockout (KO), resulting in a marked reduction in AMPK activity [40]. In addition to LKB1 serving as the primary upstream kinase for AMPK, phosphorylation, and thereby activation of AMPK, can also be induced by Ca^2+^/calmodulin-dependent protein kinase kinase (CaMKK) in response to calcium flux [39,42,43]. However, these data were not generated in skeletal muscle cells and recent findings demonstrate that CaMKK2 does not activate AMPK in skeletal muscle in mice. It is more likely that the positive outcomes in earlier studies were due to off-target impact of frequently used CaMKK inhibitors, rather than CaMKK playing a role in AMPK activation in muscle [44].

Similar to the effects of exercise and muscle contraction, administering 5-aminoimidazole-4-carboxamide 1-β-d-ribofuranoside (AICAR), the most commonly used pharmacological AMPK activator, prompts glucose uptake in skeletal muscle [45,46,47]. Additionally, PF-739, an orally administered and non-selective activator of AMPK, enhances glucose uptake in muscles and increases TBC1D1 phosphorylation at Ser231 (Ser237 in humans) in mice. Moreover, it has been shown to lower blood glucose levels in non-human primates [48]. Early studies showed that the combined stimulation of the muscle by AICAR and contraction did not result in any additive effect, compared with contraction alone. [46,47]. These findings do not allow a clear statement about the exact contribution of AMPK to contraction-stimulated glucose uptake in the skeletal muscle. There appear to be several partially overlapping pathways involved in the regulation of glucose uptake after contraction, one of which is AMPK dependent. 

The stimulatory impact of AICAR on muscle glucose uptake is completely abolished by AMPK deficiency in transgenic and knockout mouse models [42,49,50,51]. In contrast, the inactivity or depletion of the AMPK α1 or α2 subunit only reduced, but did not eliminate, contraction-induced glucose uptake in skeletal muscle [49,52]. Furthermore, the concurrent muscle-specific knockout of both regulatory AMPK β1 and β2 subunits in mice results in a decrease in glucose uptake stimulated by muscle contraction [53], ultimately emphasizing the significance of AMPK in exercise-induced glucose transport.

However, additional research presents a somewhat inconclusive view of the function of AMPK in glucose uptake stimulated by exercise. While AICAR-stimulated glucose transport was indeed abolished in knockout and transgenic mouse models of the AMPK α2 subunit, it was found that contraction-stimulated glucose uptake in vitro remained unaffected in both the extensor digitorum longus (EDL) and soleus muscle throughout these studies [50,54]. Additionally, muscle glucose uptake during in vivo exercise remained unaltered in mice with muscle-specific knockout of AMPKα1 and -α2 [55], as did muscle glucose clearance in those mice and in mice with functional depletion of AMPKα2. However, AICAR-induced glucose clearance was abrogated in the latter mouse model, indicating the presence of further contraction mediated regulators of glucose uptake, besides AMPK [55,56]. Using a mouse model with inducible skeletal muscle-specific knockout of both catalytic AMPKα1 and α2 subunits, Hingst and Kjobsted et al. investigated the direct effects of acute AMPK deficiency. This approach sidestepped any secondary effects of lifelong AMPK depletion that may occur in conventional models. They found no changes in exercise-stimulated glucose uptake in vivo or contraction-stimulated glucose uptake ex vivo across different oxidative and glycolytic skeletal muscles [57]. Furthermore, they demonstrated that AMPK knockout in glycolytic muscle eliminates the phosphorylation of TBC1D1 Ser231 induced by both contraction and treadmill exercise. This suggests an impact of AMPK on TBC1D1 that is decoupled from glucose uptake induction at first glance [57]. However, it is worth noting that only Ser231 was tested for phosphorylation, leaving room for speculation about exercise-induced glucose uptake being mediated by other TBC1D1 sites besides Ser231. It is possible that these alternate sites were targeted by kinases other than AMPK. This theory finds support in previous studies, where a mutation of TBC1D1 Ser231 (Ser231Ala) only slightly decreased muscle glucose uptake induced by AICAR, but not exercise [51]. A reduction in contraction-stimulated TBC1D1 phosphorylation at Ser231 and few other sites upon AMPK deficiency has also been demonstrated in other studies [58,59,60]. Not all phosphorylation sites that regulate skeletal muscle contraction signaling appear to have their primary function during exercise training. There is evidence to suggest that RabGAP phosphorylation sites, specifically TBC1D4-Ser711, play a role in enhancing whole-body and muscle insulin sensitivity following exercise and skeletal muscle contraction [61,62]. However, a dearth of research exists regarding the majority of phosphorylation sites of TBC1D1 when activated by muscle contraction or AMPK. We have recently reported a decrease in contraction-mediated glucose uptake in transgenic mice that overexpress the kinase-dead AMPKα1 subunit, which was not further reduced by RabGAP knockout in soleus muscle. However, in EDL muscle, further knockout of the predominant RabGAP TBC1D1 resulted in a more significant decrease in contraction-mediated glucose uptake. This observation indicates that other kinases, aside from AMPK, may induce glucose uptake by regulating TBC1D1 [63].

**Figure 1:**
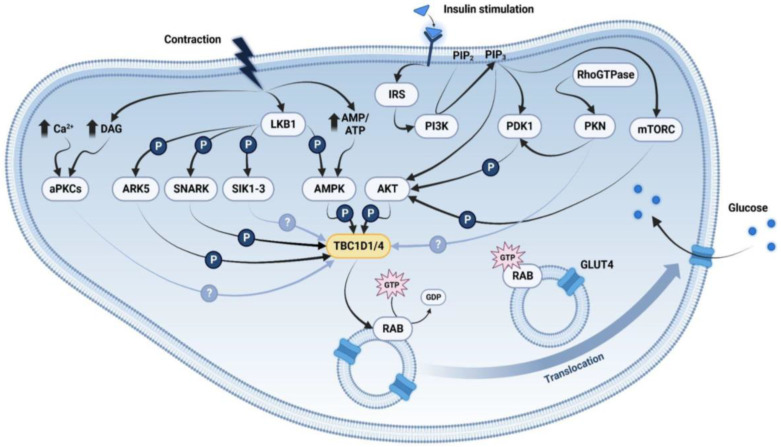
Intracellular signaling pathways for insulin- and contraction-stimulated glucose transporter 4 (GLUT4) translocation in skeletal muscle through the regulation of TBC1D1 and TBC1D4. Insulin stimulates the phosphorylation of TBC1D1/4 through activation of Akt by the upstream regulators insulin receptor substrate (IRS)-phosphatidylinositol 3-kinase (PI3K), mammalian target of rapamycin complex (mTORC) and phosphoinositide-dependent kinase-1 (PDK1), which is further regulated by protein kinase N (PKN). Contraction leads to the activation of liver kinase B1 (LKB1) and rising AMP/ATP ratio, which stimulate AMPK to phosphorylate TBC1D1/4. Besides AMPK, other AMPK-related kinases (ARKs) controlled by LKB1 are indicated to regulate TBC1D1/4 including ARK5, SNF1-ARK (SNARK) and salt inducible kinase 1-3 (SIK1-3), as well as atypical protein kinases C (aPKCs). Arrows in grey indicate suggested regulatory mechanisms. Once phosphorylated, TBC1D1/4 will no longer inhibit the translocation of GLUT4-containing vesicles through induction of RAB-bound GTP hydrolysis, allowing incorporation of GLUT4 into the plasma membrane. PIP2/3 = phosphatidylinositol-bi/triphosphate; DAG = diacylglycerol; figure created with BioRender.com.

These data suggest that AMPK plays a crucial role in regulating energy homeostasis in the muscle. Furthermore, it indicates that activation of AMPK is adequate but not imperative in stimulating glucose uptake through the phosphorylation of TBC1D1/D4 in response to contraction and exercise. Therefore, we propose that there are various, partially overlapping pathways that result in the inhibition of the RabGAPs and regulate glucose uptake following exercise, guaranteeing a proper functioning of this essential and evolutionarily conserved mechanism (Figure 1).

### 3.2. LKB1 Regulates Multiple AMPK-Related Kinases Upstream of TBC1D1 and TBC1D4

The hypothesis of redundancy in RabGAP phosphorylation by multiple kinases is supported by the results of a study that aimed at identifying upstream kinases of AMPK-regulated acetyl-coA-carboxylase 2. In this study, 18 different Ser/Thr kinases were found to be upregulated by >25% during in situ contraction of skeletal muscle from kinase-dead AMPK α2 transgenic mice [64]. A sophisticated study conducted by Hoffman and Parker et al. employed a global analysis of protein phosphorylation in human skeletal muscle biopsies subsequent to a single bout of high-intensity exercise training. The researchers found that, aside from AMPK, 44 kinases were phosphorylated at a minimum of one site [65]. These findings indicate that numerous kinases are regulated during exercise, with potential implications for the regulation of exercise-induced glucose uptake and phosphorylation of RabGAPs.

Some of these kinases could be regulated by LKB1, which acts as a master regulator for 12 AMPK-related protein kinases (ARKs) in addition to AMPK [66]. The significance of LKB1 in contraction-induced glucose uptake was shown in mice with muscle-specific knockout of LKB1 and hypomorphic LKB1 knockout mice, exhibiting severely reduced glucose uptake after in situ and in vitro contraction [40]. Additionally, another study found reduced glucose uptake and abolished phosphorylation of TBC1D4 and TBC1D1 (determined using phospho-AKT substrate (PAS) antibodies) in skeletal muscle of muscle-specific LKB1 knockout mice following in situ contraction [67]. In contrast, a different study found no impairments to exercise-induced glucose transport in LKB1-deficient mice [68]. While the role of LKB1 in the contraction-activated signaling axis is not yet fully understood, it may serve as a significant signaling hub for regulating multiple kinases involved in the modulation of GLUT4 translocation. In the subsequent sections, we will discuss the impact of five LKB1-regulated kinases—ARK5, SNARK and SIK1-3—and PKC and PKN in the control of RabGAPs and glucose uptake (Table 1).

### 3.3. SNARK and ARK5 Present Promising Upstream Kinases of TBC1D1 and TBC1D4

ARK5 (alias NUAK1) and SNF1-ARK (SNARK) (alias NUAK2), two closely related members of the ARK subfamily, have been shown to regulate exercise-stimulated glucose uptake. Koh and colleagues have demonstrated that SNARK is expressed in various types of skeletal muscle fibers and is activated by contraction and exercise in the tibialis anterior (TA) muscle of mice and by contraction alone in EDL, as well as by exercise in the vastus lateralis muscle of humans. Contraction-stimulated SNARK activity in mouse TA muscle was blunted following muscle-specific knockout of LKB1 [67]. Overexpression of a dominant negative SNARK (mtSNARK) variant in the mouse TA muscle resulted in decreased uptake of glucose stimulated by contraction that was not exacerbated by the expression of mtSNARK in AMPKα2-inactive transgenic mice. Overexpression of mtSNARK and heterozygous SNARK whole-body knockout also led to reduced TBC1D4 and TBC1D1 phosphorylation induced by contraction (as determined using PAS antibody). Furthermore, heterozygous SNARK depletion reduced glucose uptake induced by in vitro contraction in soleus muscle and induced by treadmill exercise in gastrocnemius muscle [67,70]. Insulin-stimulated glucose uptake, however, remained unaltered by the overexpression of mtSNARK or heterozygous SNARK knockout [67]. Accordingly, knockdown of SNARK expression to 40% in cultured myotubes from human vastus lateralis biopsies did not affect insulin-stimulated glucose uptake [88]. In the heart, phosphorylation of SNARK was demonstrated through ischemia and treadmill exercise. Additionally, glucose uptake induced by exercise was abolished in heterozygous SNARK knockout mice. In HL1 cardiomyocytes, knockdown of SNARK resulted in a decreased glucose uptake and ischemia-induced phosphorylation of TBC1D4 at Ser711, while insulin-stimulated TBC1D4 phosphorylation was unaffected [70]. Collectively, the data suggest that SNARK may act in a pathway downstream of LKB1 to influence exercise-induced glucose uptake in skeletal muscle and the heart, presumably via phosphorylation of the RabGAPs. However, no such role is evident for insulin-stimulated glucose uptake in these tissues. Thus, SNARK may play an important role specifically in regulating skeletal muscle exercise metabolism.

ARK5, which is closely related to SNARK, is expressed in skeletal muscle and becomes phosphorylated in response to muscle contraction, insulin stimulation and AICAR treatment [89]. However, whether its phosphorylation is sufficient to induce ARK5 kinase activity is disputed, as one study showed enhanced ARK5 activity upon phosphorylation but a later study could not confirm this (tested towards SAMS peptide) [89,90]. A study from Inazuka et al. postulated a role for ARK5 in suppressing insulin-mediated glucose uptake in skeletal muscle and reported that muscle-specific knockout of ARK5 led to increased muscle insulin sensitivity in mice. This study reported altered IRS1 phosphorylation, increased IRS1 and Akt activity, and TBC1D4 phosphorylation at Thr649 (Thr642 in human) in soleus muscle in response to glucose administration in this mouse model on a high-fat diet (HFD) [69]. However, these findings do not indicate direct interaction of ARK5 with TBC1D4. Instead, they suggest a regulation upstream of RabGAPs in the insulin signaling pathway. The impact of exercise on ARK5 activity in the regulatory pathway for glucose uptake was not investigated in this study, despite the implications of muscle contraction on ARK5 activation [69]. Koh et al. also investigated the role of ARK5 in contraction-stimulated glucose uptake but contrary to their results regarding SNARK, they found no evidence of ARK5 activation upon in situ contraction in rat muscle, and contraction-stimulated glucose uptake remained unaltered in TA muscle overexpressing mutant ARK5 [67]. In contrast, a study from our lab showed phosphorylation of TBC1D1 at least at Ser231, Ser660 and Ser700 by NUAK1 in vitro [63]. Therefore, in vivo data suggest that ARK5 plays a different role than SNARK by negatively regulating muscular insulin signaling and showing no effect on contraction-induced glucose transport. However, in vitro assays show direct activity of ARK5 towards TBC1D1. Hence, more research is required to understand which role ARK5 plays in exercise-stimulated glucose uptake and RabGAP regulation.

### 3.4. Salt Inducible Kinases (SIKs) Are Positive Regulators of Glucose Uptake and GLUT4 Levels in Adipocytes, and May Also Be Promising Factors in Skeletal Muscle Contraction Signaling

Similar to ARK5 and SNARK, the salt inducible kinases SIK1, SIK2 and SIK3 (also known as QIK and QSK, respectively) are Ser/Thr protein kinases of the ARK subfamily and activated by LKB1 [66,68]. Aside from LKB1, SIK1-3 are also regulated by cyclic AMP-dependent protein kinase. SIKs affect gene expression by phosphorylating class 2a histone deacetylases (HDACs) and CRCTs (CREB-regulated transcriptional co-activators) which activate the transcription factor CREB (cyclic AMP-response-element binding protein). Thereby, SIKs are believed to play a role in regulating a range of physiological and pathophysiological processes, such as metabolic processes, like hepatic gluconeogenesis and importantly GLUT4 expression in adipocytes as reviewed in [91]. To date, there is a shortage of data on the effect of SIKs on glucose uptake in skeletal muscle, especially in regards to muscle contraction or exercise. Verbrugge et al. recently conducted a review of datasets regarding exercise-induced gene expression and muscle glucose uptake, which identified regulation of *Sik1* and *Sik2* in mouse skeletal muscle in response to exercise, among others, with a positive association observed in glucose uptake [92]. Another study demonstrated that *Sik1* expression is elevated in skeletal muscle of mice on HFD and global deletion of SIK1 in HFD-fed mice led to improved glucose tolerance, muscle insulin sensitivity and glucose uptake in skeletal muscle. Additionally, muscle-specific knockout of SIK1 in HFD-fed mice also tends to increase insulin-stimulated glucose uptake into skeletal muscle in vivo [73]. Contrary to the limited available data on SIK function in skeletal muscle, numerous studies have examined the regulatory function of SIKs in glucose uptake and on TBC1D4 in adipose tissue [71,72,74,75]. Those data show that SIKs influence glucose uptake through regulation of the expression levels of GLUT4 due to control of CRCTs and implicate an indirect role of SIKs in the regulation of RabGAPs that is most likely due to the control of the RabGAP upstream kinase Akt, at least in adipose tissue. Currently, there is no evidence to support a direct correlation between SIKs, RabGAPs and contraction-mediated glucose uptake in skeletal muscle. However, as SIKs are direct downstream targets of LKB1 and have been shown to regulate glucose uptake and GLUT4 levels in adipocytes, these proteins may also be promising factors in skeletal muscle contraction signaling.

### 3.5. Atypical, Conventional and Novel Protein Kinase C (aPKCs, cPKCs, nPKCs) Differently Regulates Insulin- and Contraction-Stimulated Glucose Uptake

Protein kinase C (PKC) is a family of lipid-sensitive enzymes that participates in various cellular functions. The serine–threonine kinases have extensive involvement in different cellular processes by controlling the function of other proteins through phosphorylation. Various PKC isoforms were found to be upregulated and activated by muscle contraction [64] and exercise [92,93,94,95,96,97] in both mice and humans. The regulation of novel PKCs (nPKCs) (δ, ε, η, θ) depends on diacylglycerol (DAG) while conventional PKCs (cPKCs) (α, β1, β2, γ) require additional Ca2+ flux as reviewed in [98]. Atypical PKCs (aPKCs) (ζ, λ) (λ is the mouse ortholog of human PKCι) are not controlled by DAG but can be activated by ceramides and sphingosine-1-phosphate [98,99,100].

Several conventional and novel PKC isoforms have been implicated in the development of muscle insulin resistance, while some reports suggest aPKCs in positively regulating muscle glucose uptake. In mice, deficiency of the cPKC-α or cPKC-β each led to an increased insulin-stimulated glucose uptake in isolated soleus muscle and increased glucose uptake and GLUT4 translocation in isolated adipocytes [76,79]. Multiple studies in mice and humans indicate that nPKC-θ, the most abundant PKC isoform in skeletal muscle [101], negatively regulates insulin signaling by phosphorylating IRS1 at IRS1-inhibiting serine sites [80,81,102,103]. Conversely, to DAG-regulated c/nPKCs, aPKCs may play a positive role in regulating glucose uptake by insulin stimulation, as demonstrated by a study in L6 myotubes showing reduced basal- and insulin-stimulated glucose transport by overexpression of kinase-inactive aPKC-ζ [83]. In line with this, Farese et al. reported reduced insulin-stimulated glucose uptake in vastus lateralis muscle in vivo and in soleus and EDL muscle in vitro of aPKC-λ muscle-specific knockout mice [84]. Moreover, increases in GLUT4 in proximity to the plasma membrane in response to insulin stimulation were blunted in skeletal muscle of these mice [84]. The mechanism underlying the influence of aPKCs on insulin-induced glucose uptake may be related to the facilitation of membrane fusion of GSVs [104].

Besides their influence on insulin-stimulated glucose uptake in skeletal muscle, PKCs have also been suggested to be involved in contraction- and exercise-regulated glucose transport. Early studies have shown that PKC translocates intracellularly in response to contraction. This correlates with increasing concentrations of DAGs and suggests the presence of a causal relationship between contraction-induced PKC translocation and glucose transport (PMID: 2808343 and 3595854) [105,106]. Another study showed that downregulating the activity of PKCs in rat soleus muscle by phorbol ester treatment ex vivo led to a loss of contraction-mediated glucose uptake [107]. Treatment of rat soleus muscle with a PKC inhibitor reduced contraction- but not insulin-stimulated glucose uptake [108]. Inhibition of cPKC-α, the predominant cPKC isoform in skeletal muscle, attenuates contraction-stimulated glucose uptake in skeletal muscle ex vivo. However, this finding is contradicted by the results of unaffected contraction-stimulated glucose uptake after muscle-specific cPKC-α knockout. The authors explain this discrepancy by citing unspecific effects of the inhibitors used or by compensatory effects of nPKCs and aPKCs [77]. Muscle specific knockout of aPKC-λ in mice or inactivation of aPKC-λ in L6-myotubes eliminated metformin- and AICAR- or metformin- and insulin-induced glucose uptake, respectively [85]. However, the authors also showed that glucose uptake in vastus lateralis muscle stimulated by treadmill exercise was not affected in the muscle specific aPKC-λ knockout mice, although exercise was adequate to activate aPKC-λ in control animals [85]. A follow-up study even reported increased glucose transport in the soleus muscle of muscle-specific aPKC-λ knockout mice and after overexpression of the other atypical PKC aPKC-ζ in TA muscle stimulated by ex vivo contraction [86]. 

Few studies exist that suggest a role for PKCs in regulating the phosphorylation of RabGAPs. Individual knockdown of nPKC-θ and of cPKC-α blunted calcium ionophore ionomycin induced phosphorylation of TBC1D4 at site Thr642 and TBC1D1 at site Thr590 (Thr596 in humans) and inhibited GLUT4 translocation in L6 myoblasts [78]. Furthermore, stimuli that are known to activate c/nPKCs also induced TBC1D1/4 phosphorylation (determined with PAS antibody) and GLUT4 translocation in CHO-IR cells and L6 myotubes [109]. In contrast, the study by Yu et al. showed equally induced AMPK and TBC1D4 phosphorylation (determined using PAS antibody) by contraction of soleus muscle of WT and aPKC-λ mKO mice, suggesting no involvement of aPKCλ in the regulation of RabGAPs [86]. Although insulin-stimulated glucose uptake and GLUT4 translocation was impaired in muscle-specific aPKC-λ knockout mice in the study by Farese et al., TBC1D1/4 phosphorylation (determined using PAS antibody) after insulin stimulation was unaltered in vastus lateralis muscle of these aPKC-λ-deficient mice [84].

In conclusion, DAG-regulated c/nPKCs are suggested to play a role in negative feedback regulation of insulin signaling in skeletal muscle, presumably via deactivation of IRS1. However, the role of c/n PKCs during contraction may differ from the one in the insulin pathway, as the regulation on IRS1 is not as relevant in the contraction pathway. It may be the case that c/nPKCs have a positive influence on the intracellular contraction cascade. However, further non-inhibitor-based studies are necessary to confirm this, as inhibitor-based results could not be replicated in a single knockout study [77]. Further investigation is required to determine the influence on RabGAPs. Some studies have shown a direct or indirect role of c/nPKCs in phosphorylating TBC1D1/4, but the evidence is insufficient to make a definitive statement. aPKCs seem to act differently from c/nPKCs and might positively regulate insulin-stimulated glucose uptake in muscle, possibly by regulating GSV translocation [84]. However, there is currently no substantial evidence supporting the significant involvement of aPKC in contraction-stimulated glucose uptake in skeletal muscle.

### 3.6. Protein Kinase N2 (PKN2) Binds to Rac1 and May Serve as a Negative Regulator of AMPK Activity

The PKN family, also referred to as protein kinase C-related kinases (PRK), because of their catalytic domain similar to that of protein kinase C (PKC) family members, comprises three isoforms: PKN1 (or PRK1 or PKNα), PKN2 (or PRK2 or PKNγ) and PKN3 (or PRK3 or PKNβ). PKNs are bound by small Rho GTPases such as Rac1 [110,111] which leads to a conformational change in PKN that enables the binding of PDK1 [112], an upstream regulator of Akt [113], both crucial in the regulation of GLUT4 translocation and insulin-regulated glucose transport (recently reviewed in [114,115]). Additionally, Rac1 is a well-known regulator of contraction-stimulated glucose uptake in skeletal muscle (PMID: 23274900) [116].

Whereas PKN1 seems to be the relevant PKN isoform in adipocytes, it has been shown that PKN2 knockdown in vitro in PHMCs (primary human muscle cells) and in vivo in mouse TA muscle both decreased insulin-stimulated glucose uptake. Interestingly, phosphorylation of AMPK and its downstream targets were increased in both models and basal- and insulin-stimulated phosphorylation of TBC1D4 at site Ser314 was enhanced in PHMCs after PKN2 knockdown [87].

Additional research is necessary to determine the precise role of PKN2 in skeletal muscle, particularly in relation to contraction-stimulated glucose transport. However, PKN2 may play a significant role in skeletal muscle contraction signaling, despite being poorly described.

### 3.7. Hypoxia Influences Skeletal Muscle Glucose Uptake through Mechanisms That Partly Overlap with Contraction and Are Independent of Insulin Signaling, including a Major Role of HIF1α

Hypoxia indicates an imbalance in tissue oxygen levels. When exercise and hypoxia coincide, the oxygen balance in skeletal muscle may be further disrupted (reviewed in [117]). Hypoxia has also been shown to affect insulin sensitivity and glucose uptake in skeletal muscle, acting as a cellular signaling mechanism. This section aims to provide a concise overview of the various implications of hypoxia on GLUT4 translocation in skeletal muscle, particularly during exercise. 

Studies in rats and humans indicate that hypoxia stimulates glucose uptake specifically in the skeletal muscle as this outcome has not yet been described for other tissues [118,119]. Due to the role of skeletal muscle glucose uptake as a critical determinant of insulin sensitivity and progression of metabolic diseases, hypoxia is also in the focus of diabetes research. When examining acute exercise in hypoxia, it was reported that insulin sensitivity improved after hypoxic exercise compared with normoxic exercise in persons with type 2 diabetic [120]. However, a beneficial impact of acute environmental (resting) hypoxia on postprandial glucose response and GLUT4 translocation has also been shown in skeletal muscle of healthy subjects [121]. Studies suggest that hypoxic training may have positive effects on skeletal muscle glucose uptake through signaling pathways similar to those activated by contraction, such as elevated Ca^2+^ concentration [119,122] or increased AMP/ATP ratio and AMPK activation [49,123]. This is supported by reports that hypoxia and contraction do not exert their stimulatory effects on glucose uptake in an additive manner [49,119,124]. However, in one of those reports, the stimulatory effect of muscle contraction by electrical stimulation was inhibited by wortmannin and calphostin C but hypoxia-induced skeletal muscle glucose uptake was unaffected by these drugs, indicating independent pathways [124]. Conversely, De Groote et al. reported unaltered activation of the AMPK-TBC1D1/4 and Akt-TBC1D1/4 axis in vastus lateralis muscle after hypoxic and normoxic exercise in healthy and pre-diabetic men [125]. Besides the intracellular contraction pathway, independency of hypoxia-stimulated muscle glucose uptake from intramuscular insulin signaling is indicated, as Mackenzie et al. demonstrated additive effects of exercise and hypoxia on insulin sensitivity in type 2 diabetic patients [120]. Furthermore, the study by Hulst et al. reported that the increases in muscle GLUT4 translocation were independent from intramuscular insulin signaling as Akt-dependent phosphorylation of TBC1D4 at Thr642 and Ser588 was mostly unchanged between hypoxic and normoxic conditions [121]. 

One factor that clearly distinguishes hypoxia from contraction and insulin stimulation is the activation of the transcription factor hypoxia-inducible factor (HIF) 1α. HIF1 is a master regulator of oxygen homeostasis which is degraded under normoxic conditions, but activated when oxygen concentrations fall (reviewed in [126]. It regulates multiple cellular responses such as angiogenesis, apoptosis and glucose metabolism [127]. HIF1α appears to have a positive effect on TBC1D4 phosphorylation and GLUT4 translocation mediated by AKT and AMPK- in C2C12 myotubes. This effect is most likely due to the regulation of genes that affect RabGAP regulation as HIF1α and TBC1D4 do not directly interact [128]. In accordance, a later study from our lab found increased insulin- and contraction-stimulated glucose uptake and TBC1D4 Thr642 phosphorylation after hypoxic treatment of human myotubes, which was abrogated after HIF1α silencing. The study also identified *RAB20* as a gene upregulated by HIF1α and linked with GLUT4 transport. Additionally, *TXNIP* was found to be a gene downregulated by HIF1α and negatively associated with Akt and TBC1D4 phosphorylation [129]. However, it is possible that this pathway is specific to skeletal muscle, as HIF1α and HIF2α were found to have a negative impact on TBC1D1 phosphorylation in 3T3-L1 adipocytes [130]. Furthermore, it should be noted that not all studies have reported an increase in the expression of HIF1α during hypoxic exercise in humans. This discrepancy could be attributed to differences in the hypoxic dose and duration used in each study [125]. 

Taken together, multiple studies showed increased hypoxia-stimulated glucose uptake specifically in skeletal muscle that is distinct from insulin signaling and partially overlaps with intramuscular signaling activated by contraction. An implication of TBC1D1/4 regulation through hypoxia seems unlikely, besides the effects by altered gene expression induced by HIF1α.

## 4. Conclusions

Multiple pathways regulate skeletal muscle glucose uptake as an evolutionarily important and highly conserved mechanism for maintaining blood glucose balance and meeting the need for enhanced energy supply during situations of stress and physical exertion. To ensure the functionality of this mechanism, many of the underlying pathways are partially functionally redundant. In this review, we have summarized the current state of knowledge on the two RabGAPs, TBC1D1 and TBC1D4, in regulating contraction-mediated glucose uptake in skeletal muscle, focusing not only on the classical regulators such as AMPK, but also on the wide field of AMPK-related kinases (ARKs) and physiologically broader stimulators such as hypoxia.

The two RabGAPs, TBC1D1 and TBC1D4, are well-known regulators of GLUT4-dependent glucose uptake through control of GSV translocation. As direct downstream phosphorylation targets of AKT and AMPK, TBC1D1 and TBC1D4 represent a key signaling hub where insulin and contraction signaling converge in skeletal muscle. However, the view that Akt and AMPK are the sole regulators of insulin- and contraction-stimulated glucose uptake is outdated. With increasing reports of insulin-sensitizing effects of exercise in recent decades, understanding the cellular pathways underlying these beneficial outcomes is essential. Future research should therefore focus on unraveling the complex network of skeletal muscle contractile responses to enable more effective prevention and treatment of metabolic disorders. 

## Figures and Tables

**Table 1:** Effects of AMPK-related kinases on glucose uptake, GLUT4 translocation, RabGAP phosphorylation and insulin signaling axis.

Kinase	Stimulus/Model	Observation	References
**AMPK**	AICAR treatment in rat	Increased glucose uptake in hindlimb muscles in vivo	[45,46]
		Increased glucose uptake in epitrochlearis ex vivo	[47]
	PF-739 treatment in: - mice - Cynomolgus monkeys	- Lowered blood glucose levels - Increased glucose uptake in EDL - Increased TBC1D1 Ser237 phosphorylation in EDL and gastrocnemius	[48]
		Lowered blood glucose levels	[48]
	Muscle AMPKα2 KD mice	Reduced contraction-stimulated glucose uptake in EDL and soleus ex vivo	[49]
		No effect on contraction-stimulated glucose transport in EDL in vitro and in EDL, TA, gastrocnemius muscle in vivo	[50]
	Muscle AMPKα1 KD or KO mice	Reduced twitch-contraction-stimulated glucose uptake soleus ex vivo	[52]
	Simultaneous muscle AMPKβ1 and β2 KO in mice	Reduced contraction-stimulated glucose uptake in EDL and soleus ex vivo	[53]
	Muscle AMPKα1 KO mice	No reduction of contraction stimulated glucose uptake in EDL in vitro	[54]
	Muscle AMPKα2 KO mice	No reduction of contraction stimulated glucose uptake in EDL and soleus in vitro	[54]
	Simultaneous muscle AMPKα1 and α2 KO in mice	- No reduction of treadmill exercise-induced glucose uptake and unchanged glucose clearance in EDL, soleus, TA, quadriceps in vivo - Abolished TBC1D1 Ser231 phosphorylation after exercise in soleus and quadriceps	[55]
	Muscle AMPKα2 KD mice	- No reduction of treadmill exercise-induced glucose clearance in soleus, gastrocnemius, quadriceps in vivo - Increased surface membrane GLUT4 content after exercise normalized to GLUT4 abundance	[56]
	Inducible simultaneous KO of muscle AMPKα1 and α2 KO in mice	- Unchanged treadmill exercise-stimulated glucose uptake in EDL, soleus, TA, quadriceps in vivo - Unchanged contraction-stimulated glucose uptake in EDL and soleus ex vivo - Abolished TBC1D1 Ser231 phosphorylation after exercise in quadriceps and after contraction in EDL	[57]
	AMPKα2 KO mice	- Reduced TBC1D1 Ser231, Thr590 and Ser600 phosphorylation after contraction in EDL ex vivo - Reduced TBC1D4 Ser588 and Thr704 phosphorylation after contraction in soleus ex vivo	[58]
	AMPKα2 KD mice	Abolished TBC1D1 Thr590 and Ser700 phosphorylation after contraction in EDL ex vivo	[58]
	Muscle AMPKα2 KD mice	Reduced TBC1D1 S231, S660 and S700 phosphorylation after contraction in TA in vivo	[60]
	Muscle AMPKα2 KD mice	Abolished TBC1D1 Ser231 and Thr590 phosphorylation after contraction in EDL ex vivo	[59]
**ARK5**	Kinase assay	Phosphorylation of TBC1D1 Ser231, Ser660 and Ser700 in vitro	[63]
	TA muscle mtARK5 mice	Unchanged contraction-stimulated glucose uptake in TA in situ	[67]
	Muscle specific ARK5 KO mice	- Increased insulin-stimulated glucose uptake in soleus ex vivo - Increased phosphorylation of TBC1D4 Thr649 in soleus after glucose administration in mice on HFD	[69]
**SNARK**	TA Muscle mtSNARK mice	- Reduced contraction-stimulated glucose uptake in TA in situ - Reduced TBC1D1 and TBC1D4 phosphorylation in TA in situ	[67]
	Heterozygous SNARK KO mice	- Reduced contraction-stimulated glucose uptake in soleus in vitro - Reduced TBC1D1 and TBC1D4 phosphorylation after contraction in soleus in vitro	[67]
	Heterozygous SNARK KO mice	- Reduced treadmill exercise-stimulated glucose uptake in gastrocnemius and heart	[67,70]
	HL1 (mouse cardio-myocyte cell line) SNARK knockdown	- Reduced ischemia-induced glucose uptake HL1 cells - Reduced ischemia-induced TBC1D4 Ser711 phosphorylation in HL1 cells	[70]
**SIK1**	*Sik1-3* silencing in human adipocytes	Reduced basal- and insulin-stimulated glucose uptake	[71]
	SIK inhibition in human adipocytes with - HG-9-91-01 - YKL-05-099	- Reduced basal- and insulin-stimulated glucose uptake (Inhibitor: YKL-05-099 and HG-9-91-01) - Reduced insulin-stimulated TBC1D4 Thr642 phosphorylation (Inhibitor: HG-9-91-01)	[72]
	- SIK1 KO mice on HFD - Muscle specific SIK1 KO mice on HFD	- Increased insulin-stimulated glucose uptake in quadriceps in vivo - Tendentially increased skeletal muscle glucose uptake in vivo	[73]
**SIK2**	mtSIK2 expression in primary rat adipocytes	Reduced insulin-stimulated glucose uptake	[74]
	OE of SIK2 in primary rat adipocytes	Increased basal glucose uptake	[74]
	Primary adipocytes of SIK2 KO mice >20 weeks old	Reduced basal- and insulin-stimulated glucose uptake	[72]
	SIK2 KO mice	- Reduced glucose and insulin tolerance (HFD and ND) - Reduced insulin-stimulated glucose uptake in skeletal muscles ex vivo	[75]
	SIK2 knockdown in 3T3-L1 pre-adipocytes	Reduced GLUT4 expression and insulin-stimulated glucose uptake (after differentiation)	[75]
	Primary adipocytes of SIK2 KO mice	- Reduced Akt and TBC1D4 phosphorylation - Reduced GLUT4 expression and glucose uptake	[75]
	SIK2 overexpression in 3T3-L1 adipocytes	- Increased basal- and insulin-stimulated Akt and TBC1D4 phosphorylation - Increased GLUT4 expression and basal- and insulin-stimulated glucose uptake	[75]
	*Sik1-3* silencing in human adipocytes	Reduced basal- and insulin-stimulated glucose uptake	[71]
	SIK inhibition in human adipocytes with - HG-9-91-01 - YKL-05-099	- Reduced basal- and insulin-stimulated glucose uptake (Inhibitor: YKL-05-099 and HG-9-91-01) - Reduced insulin-stimulated TBC1D4 Thr642 phosphorylation (Inhibitor: HG-9-91-01)	[72]
**SIK3**	*Sik1-3* silencing in human adipocytes	Reduced basal- and insulin-stimulated glucose uptake	[71]
	SIK inhibition in human adipocytes with - HG-9-91-01 - YKL-05-099	- Reduced basal- and insulin-stimulated glucose uptake (Inhibitor: YKL-05-099 and HG-9-91-01) - Reduced insulin-stimulated TBC1D4 Thr642 phosphorylation (Inhibitor: HG-9-91-01)	[72]
**cPKC**	cPKC-α KO in mice	- Increased basal- and insulin-stimulated glucose uptake in adipocytes and soleus muscle ex vivo - Increased basal- and insulin-stimulated GLUT4 translocation in adipocytes	[76]
	cPKC-α inhibition in mouse muscle - Calphostin C - Gö-6976 - Gö-6983	- Reduced contraction-stimulated glucose uptake in EDL and soleus muscle in vitro (Inhibitor: Calphostin C, Gö-6976, Gö-6983) - Reduced insulin-stimulated glucose uptake in EDL and soleus muscle in vitro (Inhibitor: Gö-6976, Gö-6983)	[77]
	cPKC-α knockdown in L6-myotubes	Reduced ionomycin-stimulated phosphorylation of TBC1D1 Thr590 and TBC1D4 Thr642	[78]
	Muscle specific cPKC-α KO in mice	Unchanged contraction-stimulated glucose uptake in EDL and soleus muscle in vitro	[77]
	cPKC-β KO in mice	- Increased insulin-stimulated glucose uptake in adipocytes and soleus muscle in vitro - Increased insulin-stimulated GLUT4 translocation in adipocytes	[79]
**nPKC**	nPKC-θ KO mice	Rescued impaired insulin-stimulated glucose uptake and IRS1-Tyr phosphorylation in gastrocnemius muscle by lipid infusion in vivo	[80]
	Muscle specific nPKC-θ KO mice on HFD	Rescued impaired insulin-stimulated Akt phosphorylation in gastrocnemius muscle after HFD	[81]
	nPKC-θ knockdown in L6-myotubes	Reduced ionomycin-stimulated phosphorylation of TBC1D1 Thr590 and TBC1D4 Thr642	[78]
	Muscle nPKC-θ KD expression in mice	Reduced insulin-stimulated Akt phosphorylation	[82]
**aPKC**	aPKC-ζ KD expression in L6-myotubes	Reduced basal- and insulin-stimulated glucose uptake	[83]
	Muscle specific aPKC-ζ KO mice	- Reduced insulin-stimulated glucose uptake in vastus lateralis muscle in vivo - Reduced insulin-stimulated glucose uptake in soleus and EDL muscle in vitro - Reduced insulin-stimulated GLUT4 translocation in vastus lateralis and gastrocnemius muscle - Unaltered insulin-stimulated phosphorylation of TBC1D1/4 in vastus lateralis muscle	[84]
	Muscle specific aPKC-λ KO mice	Reduced ACIAR- and metformin-induced glucose uptake in vastus lateralis muscle in vivo and AICAR stimulation in EDL muscle ex vivo	[85]
	aPKC-λ expression in L6-myotubes	Reduced insulin- or metformin-induced glucose uptake	[85]
	aPKC-λ knockdown in L6-myotubes	Reduced insulin- or metformin-induced glucose uptake	[85]
	Muscle specific aPKC-λ KO mice	No reduction of treadmill exercise-induced glucose uptake in vastus lateralis muscle in vivo	[85]
	Muscle specific aPKC-λ KO mice	Increased contraction-stimulated glucose uptake and TBC1D4 phosphorylation in soleus muscle in vitro	[86]
	TA muscle specific T410A-aPKC-ζ KD overexpression	Increased contraction-stimulated glucose uptake in TA muscle in situ	[86]
**PKN**	PKN2 knockdown in PHMC and mouse TA muscle	- Reduced insulin-stimulated glucose uptake in PHMC in vitro and in TA muscle in vivo - No change in insulin stimulated Akt phosphorylation in PHMC in vitro - Increased AMPK and TBC1D1 Ser314 phosphorylation in PHMC in vitro	[87]

Observations highlighted in **blue** indicate a role of the respective kinase in glucose uptake/GLUT4 translocation/RabGAP regulation/regulation of insulin axis. Observations highlighted in **red** indicate negative regulation. Observations written in black letters indicate no role in the respective mechanism. KD = kinase dead, KO = knockout, TA = tibialis anterior, OE = overexpression, mt = mutated.

## References

[1] Röder P.V., Wu B., Liu Y., Han W. (2016). Pancreatic regulation of glucose homeostasis. Exp. Mol. Med..

[2] Ahlborg G., Felig P., Hagenfeldt L., Hendler R., Wahren J. (1974). Substrate turnover during prolonged exercise in man. Splanchnic and leg metabolism of glucose, free fatty acids, and amino acids. J. Clin. Investig..

[3] DeFronzo R.A., Jacot E., Jequier E., Maeder E., Wahren J., Felber J.P. (1981). The effect of insulin on the disposal of intravenous glucose. Results from indirect calorimetry and hepatic and femoral venous catheterization. Diabetes.

[4] Martin B.C., Warram J.H., Krolewski A.S., Soeldner J.S., Kahn C.R., Bergman R.N. (1992). Role of glucose and insulin resistance in development of type 2 diabetes mellitus: Results of a 25-year follow-up study. Lancet.

[5] Haffner S.M., Miettinen H., Gaskill S.P., Stern M.P. (1995). Decreased insulin secretion and increased insulin resistance are independently related to the 7-year risk of NIDDM in Mexican-Americans. Diabetes.

[6] Abdul-Ghani M.A., Tripathy D., Defronzo R.A. (2006). Contributions of beta-cell dysfunction and insulin resistance to the pathogenesis of impaired glucose tolerance and impaired fasting glucose. Diabetes Care.

[7] Frank R.N. (2004). Diabetic Retinopathy. N. Engl. J. Med..

[8] Dronavalli S., Duka I., Bakris G.L. (2008). The pathogenesis of diabetic nephropathy. Nat. Clin. Pract. Endocrinol. Metab..

[9] Li W., Zhang X., Sang H., Zhou Y., Shang C., Wang Y., Zhu H. (2019). Effects of hyperglycemia on the progression of tumor diseases. J. Exp. Clin. Cancer Res..

[10] Laakso M. (1999). Hyperglycemia and cardiovascular disease in type 2 diabetes. Diabetes.

[11] Milicevic Z., Raz I., Beattie S.D., Campaigne B.N., Sarwat S., Gromniak E., Kowalska I., Galic E., Tan M., Hanefeld M. (2008). Natural history of cardiovascular disease in patients with diabetes: Role of hyperglycemia. Diabetes Care.

[12] Mbata O., El-Magd N.F.A., El-Remessy A.B. (2017). Obesity, metabolic syndrome and diabetic retinopathy: Beyond hyperglycemia. World J. Diabetes.

[13] Grisold A., Callaghan B.C., Feldman E.L. (2017). Mediators of diabetic neuropathy: Is hyperglycemia the only culprit?. Curr. Opin. Endocrinol. Diabetes Obes..

[14] Yagihashi S., Mizukami H., Sugimoto K. (2011). Mechanism of diabetic neuropathy: Where are we now and where to go?. J. Diabetes Investig..

[15] Martin I.K., Katz A., Wahren J. (1995). Splanchnic and muscle metabolism during exercise in NIDDM patients. Am. J. Physiol..

[16] Rose A.J., Richter E.A. (2005). Skeletal muscle glucose uptake during exercise: How is it regulated?. Physiology.

[17] Kemps H., Kränkel N., Dörr M., Moholdt T., Wilhelm M., Paneni F., Serratosa L., Solberg E.E., Hansen D., Halle M. (2019). Exercise training for patients with type 2 diabetes and cardiovascular disease: What to pursue and how to do it. A Position Paper of the European Association of Preventive Cardiology (EAPC). Eur. J. Prev. Cardiol..

[18] Taylor E.B., An D., Kramer H.F., Yu H., Fujii N.L., Roeckl K.S.C., Bowles N., Hirshman M.F., Xie J., Feener E.P. (2008). Discovery of TBC1D1 as an insulin-, AICAR-, and contraction-stimulated signaling nexus in mouse skeletal muscle. J. Biol. Chem..

[19] Kramer H.F., Witczak C.A., Taylor E.B., Fujii N., Hirshman M.F., Goodyear L.J. (2006). AS160 regulates insulin- and contraction-stimulated glucose uptake in mouse skeletal muscle. J. Biol. Chem..

[20] Kramer H.F., Witczak C.A., Fujii N., Jessen N., Taylor E.B., Arnolds D.E., Sakamoto K., Hirshman M.F., Goodyear L.J. (2006). Distinct signals regulate AS160 phosphorylation in response to insulin, AICAR, and contraction in mouse skeletal muscle. Diabetes.

[21] Mafakheri S., Chadt A., Al-Hasani H. (2018). Regulation of RabGAPs involved in insulin action. Biochem. Soc. Trans..

[22] Espelage L., Al-Hasani H., Chadt A. (2020). RabGAPs in skeletal muscle function and exercise. J. Mol. Endocrinol..

[23] Joost H.-G., Bell G.I., Best J.D., Birnbaum M.J., Charron M.J., Chen Y.T., Doege H., James D.E., Lodish H.F., Moley K.H. (2002). Nomenclature of the GLUT/SLC2A family of sugar/polyol transport facilitators. Am. J. Physiol. Endocrinol. Metab..

[24] Marette A., Burdett E., Douen A., Vranic M., Klip A. (1992). Insulin induces the translocation of GLUT4 from a unique intracellular organelle to transverse tubules in rat skeletal muscle. Diabetes.

[25] Lauritzen H.P. (2013). Insulin- and contraction-induced glucose transporter 4 traffic in muscle: Insights from a novel imaging approach. Exerc. Sport. Sci. Rev..

[26] Taniguchi C.M., Emanuelli B., Kahn C.R. (2006). Critical nodes in signalling pathways: Insights into insulin action. Nat. Rev. Mol. Cell Biol..

[27] Kane S., Sano H., Liu S.C., Asara J.M., Lane W.S., Garner C.C., Lienhard G.E. (2002). A method to identify serine kinase substrates. Akt phosphorylates a novel adipocyte protein with a Rab GTPase-activating protein (GAP) domain. J. Biol. Chem..

[28] Stenmark H. (2009). Rab GTPases as coordinators of vesicle traffic. Nat. Rev. Mol. Cell Biol..

[29] Sano H., Kane S., Sano E., Mîinea C.P., Asara J.M., Lane W.S., Garner C.W., Lienhard G.E. (2003). Insulin-stimulated phosphorylation of a Rab GTPase-activating protein regulates GLUT4 translocation. J. Biol. Chem..

[30] Homma Y., Hiragi S., Fukuda M. (2021). Rab family of small GTPases: An updated view on their regulation and functions. FEBS J..

[31] Mîinea C.P., Sano H., Kane S., Sano E., Fukuda M., Peränen J., Lane W.S., Lienhard G.E. (2005). AS160, the Akt substrate regulating GLUT4 translocation, has a functional Rab GTPase-activating protein domain. Biochem. J..

[32] Larance M., Ramm G., Stöckli J., van Dam E.M., Winata S., Wasinger V., Simpson F., Graham M., Junutula J.R., Guilhaus M. (2005). Characterization of the role of the Rab GTPase-activating protein AS160 in insulin-regulated GLUT4 trafficking. J. Biol. Chem..

[33] Sun Y., Bilan P.J., Liu Z., Klip A. (2010). Rab8A and Rab13 are activated by insulin and regulate GLUT4 translocation in muscle cells. Proc. Natl. Acad. Sci. USA.

[34] Zhou Z., Menzel F., Benninghoff T., Chadt A., Du C., Holman G.D., Al-Hasani H. (2017). Rab28 is a TBC1D1/TBC1D4 substrate involved in GLUT4 trafficking. FEBS Lett..

[35] Eickelschulte S., Hartwig S., Leiser B., Lehr S., Joschko V., Chokkalingam M., Chadt A., Al-Hasani H. (2021). AKT/AMPK-mediated phosphorylation of TBC1D4 disrupts the interaction with insulin-regulated aminopeptidase. J. Biol. Chem..

[36] Kaddai V., Le Marchand-Brustel Y., Cormont M. (2008). Rab proteins in endocytosis and Glut4 trafficking. Acta Physiol..

[37] Chavez J.A., Roach W.G., Keller S.R., Lane W.S., Lienhard G.E. (2008). Inhibition of GLUT4 translocation by Tbc1d1, a Rab GTPase-activating protein abundant in skeletal muscle, is partially relieved by AMP-activated protein kinase activation. J. Biol. Chem..

[38] Hawley S.A., Pan D.A., Mustard K.J., Ross L., Bain J., Edelman A.M., Frenguelli B.G., Hardie D.G. (2005). Calmodulin-dependent protein kinase kinase-beta is an alternative upstream kinase for AMP-activated protein kinase. Cell Metab..

[39] Hawley S.A., Boudeau J., Reid J.L., Mustard K.J., Udd L., Mäkelä T.P., Alessi D.R., Hardie D.G. (2003). Complexes between the LKB1 tumor suppressor, STRAD alpha/beta and MO25 alpha/beta are upstream kinases in the AMP-activated protein kinase cascade. J. Biol..

[40] Sakamoto K., McCarthy A., Smith D., Green K.A., Hardie D.G., Ashworth A., Alessi D.R. (2005). Deficiency of LKB1 in skeletal muscle prevents AMPK activation and glucose uptake during contraction. EMBO J..

[41] Oakhill J.S., Scott J.W., Kemp B.E. (2012). AMPK functions as an adenylate charge-regulated protein kinase. Trends Endocrinol. Metab..

[42] Hurley R.L., Anderson K.A., Franzone J.M., Kemp B.E., Means A.R., Witters L.A. (2005). The Ca^2+^/calmodulin-dependent protein kinase kinases are AMP-activated protein kinase kinases. J. Biol. Chem..

[43] Woods A., Dickerson K., Heath R., Hong S.P., Momcilovic M., Johnstone S.R., Carlson M., Carling D. (2005). Ca^2+^/calmodulin-dependent protein kinase kinase-beta acts upstream of AMP-activated protein kinase in mammalian cells. Cell Metab..

[44] Negoita F., Addinsall A.B., Hellberg K., Bringas C.F., Hafen P.S., Sermersheim T.J., Agerholm M., Lewis C.T., Ahwazi D., Ling N.X. (2023). CaMKK2 is not involved in contraction-stimulated AMPK activation and glucose uptake in skeletal muscle. Mol. Metab..

[45] Merrill G.F., Kurth E.J., Hagberg J.M., Coyle E.F., Baldwin K.M., Cartee G.D., Fontana L., Joyner M.J., Kirwan J.P., Seals D.R. (1997). AICA riboside increases AMP-activated protein kinase, fatty acid oxidation, and glucose uptake in rat muscle. Am. J. Physiol..

[46] Bergeron R., Russell R.R., Young L.H., Marcucci M., Lee A., Shulman G.I., Shaw A., Jeromson S., Watterson K.R., Pediani J.D. (1999). Effect of AMPK activation on muscle glucose metabolism in conscious rats. Am. J. Physiol..

[47] Hayashi T., Hirshman M.F., Kurth E.J., Winder W.W., Goodyear L.J. (1998). Evidence for 5′ AMP-activated protein kinase mediation of the effect of muscle contraction on glucose transport. Diabetes.

[48] Cokorinos E.C., Delmore J., Reyes A.R., Albuquerque B., Kjøbsted R., Jørgensen N.O., Tran J.-L., Jatkar A., Cialdea K., Esquejo R.M. (2017). Activation of Skeletal Muscle AMPK Promotes Glucose Disposal and Glucose Lowering in Non-human Primates and Mice. Cell Metab..

[49] Mu J., Brozinick J.T., Valladares O., Bucan M., Birnbaum M.J. (2001). A role for AMP-activated protein kinase in contraction- and hypoxia-regulated glucose transport in skeletal muscle. Mol. Cell.

[50] Fujii N., Hirshman M.F., Kane E.M., Ho R.C., Peter L.E., Seifert M.M., Goodyear L.J. (2005). AMP-activated protein kinase alpha2 activity is not essential for contraction- and hyperosmolarity-induced glucose transport in skeletal muscle. J. Biol. Chem..

[51] Chen Q., Xie B., Zhu S., Rong P., Sheng Y., Ducommun S., Chen L., Quan C., Li M., Sakamoto K. (2017). A Tbc1d1 (Ser231Ala)-knockin mutation partially impairs AICAR- but not exercise-induced muscle glucose uptake in mice. Diabetologia.

[52] Jensen T.E., Schjerling P., Viollet B., Wojtaszewski J.F., Richter E.A. (2008). AMPK alpha1 activation is required for stimulation of glucose uptake by twitch contraction, but not by H2O2, in mouse skeletal muscle. PLoS ONE.

[53] O’Neill H.M., Maarbjerg S.J., Crane J.D., Jeppesen J., Jørgensen S.B., Schertzer J.D., Shyroka O., Kiens B., van Denderen B.J., Tarnopolsky M.A. (2011). AMP-activated protein kinase (AMPK) beta1beta2 muscle null mice reveal an essential role for AMPK in maintaining mitochondrial content and glucose uptake during exercise. Proc. Natl. Acad. Sci. USA.

[54] Jørgensen S.B., Viollet B., Andreelli F., Frøsig C., Birk J.B., Schjerling P., Vaulont S., Richter E.A., Wojtaszewski J.F.P. (2004). Knockout of the alpha2 but not alpha1 5′-AMP-activated protein kinase isoform abolishes 5-aminoimidazole-4-carboxamide-1-beta-4-ribofuranosidebut not contraction-induced glucose uptake in skeletal muscle. J. Biol. Chem..

[55] Fentz J., Kjøbsted R., Birk J.B., Jordy A.B., Jeppesen J., Thorsen K., Schjerling P., Kiens B., Jessen N., Viollet B. (2015). AMPKalpha is critical for enhancing skeletal muscle fatty acid utilization during in vivo exercise in mice. FASEB J..

[56] Maarbjerg S.J., Jørgensen S.B., Rose A.J., Jeppesen J., Jensen T.E., Treebak J.T., Birk J.B., Schjerling P., Wojtaszewski J.F.P., Richter E.A. (2009). Genetic impairment of AMPKalpha2 signaling does not reduce muscle glucose uptake during treadmill exercise in mice. Am. J. Physiol. Endocrinol. Metab..

[57] Hingst J.R., Kjøbsted R., Birk J.B., Jørgensen N.O., Larsen M.R., Kido K., Larsen J.K., Kjeldsen S.A., Fentz J., Frøsig C. (2020). Inducible deletion of skeletal muscle AMPKalpha reveals that AMPK is required for nucleotide balance but dispensable for muscle glucose uptake and fat oxidation during exercise. Mol. Metab..

[58] Treebak J.T., Pehmøller C., Kristensen J.M., Kjøbsted R., Birk J.B., Schjerling P., Richter E.A., Goodyear L.J., Wojtaszewski J.F.P. (2014). Acute exercise and physiological insulin induce distinct phosphorylation signatures on TBC1D1 and TBC1D4 proteins in human skeletal muscle. J. Physiol..

[59] Pehmøller C., Treebak J.T., Birk J.B., Chen S., MacKintosh C., Hardie D.G., Richter E.A., Wojtaszewski J.F.P., Hargreaves M., Szekeres F. (2009). Genetic disruption of AMPK signaling abolishes both contraction- and insulin-stimulated TBC1D1 phosphorylation and 14-3-3 binding in mouse skeletal muscle. Am. J. Physiol. Endocrinol. Metab..

[60] Vichaiwong K., Purohit S., An D., Toyoda T., Jessen N., Hirshman M.F., Goodyear L.J. (2010). Contraction regulates site-specific phosphorylation of TBC1D1 in skeletal muscle. Biochem. J..

[61] Jørgensen N.O., Wojtaszewski J.F.P., Kjøbsted R. (2018). Serum Is Not Necessary for Prior Pharmacological Activation of AMPK to Increase Insulin Sensitivity of Mouse Skeletal Muscle. Int. J. Mol. Sci..

[62] Kjøbsted R., Kristensen J.M., Eskesen N.O., Kido K., Fjorder K., Damgaard D.F., Larsen J.K., Andersen N.R., Birk J.B., Gudiksen A. (2023). TBC1D4-S711 Controls Skeletal Muscle Insulin Sensitization After Exercise and Contraction. Diabetes.

[63] de Wendt C., Espelage L., Eickelschulte S., Springer C., Toska L., Scheel A., Bedou A.D., Benninghoff T., Cames S., Stermann T. (2021). Contraction-Mediated Glucose Transport in Skeletal Muscle Is Regulated by a Framework of AMPK, TBC1D1/4, and Rac1. Diabetes.

[64] Dzamko N., Schertzer J.D., Ryall J.G., Steel R., Macaulay S.L., Wee S., Chen Z., Michell B.J., Oakhill J.S., Watt M.J. (2008). AMPK-independent pathways regulate skeletal muscle fatty acid oxidation. J. Physiol..

[65] Hoffman N.J., Parker B.L., Chaudhuri R., Fisher-Wellman K.H., Kleinert M., Humphrey S.J., Yang P., Holliday M., Trefely S., Fazakerley D.J. (2015). Global Phosphoproteomic Analysis of Human Skeletal Muscle Reveals a Network of Exercise-Regulated Kinases and AMPK Substrates. Cell Metab..

[66] Lizcano J.M., Göransson O., Toth R., Deak M., Morrice N.A., Boudeau J., Hawley S.A., Udd L., Makela T.P., Hardie D.G. (2004). LKB1 is a master kinase that activates 13 kinases of the AMPK subfamily, including MARK/PAR-1. EMBO J..

[67] Koh H.-J., Toyoda T., Fujii N., Jung M.M., Rathod A., Middelbeek R.J.-W., Lessard S.J., Treebak J.T., Tsuchihara K., Esumi H. (2010). Sucrose nonfermenting AMPK-related kinase (SNARK) mediates contraction-stimulated glucose transport in mouse skeletal muscle. Proc. Natl. Acad. Sci. USA.

[68] Jeppesen J., Maarbjerg S.J., Jordy A.B., Fritzen A.M., Pehmøller C., Sylow L., Serup A.K., Jessen N., Thorsen K., Prats C. (2013). LKB1 regulates lipid oxidation during exercise independently of AMPK. Diabetes.

[69] Inazuka F., Sugiyama N., Tomita M., Abe T., Shioi G., Esumi H. (2012). Muscle-specific knock-out of NUAK family SNF1-like kinase 1 (NUAK1) prevents high fat diet-induced glucose intolerance. J. Biol. Chem..

[70] Sun X., Lessard S.J., An D., Koh H., Esumi H., Hirshman M.F., Goodyear L.J. (2019). Sucrose nonfermenting AMPK-related kinase (SNARK) regulates exercise-stimulated and ischemia-stimulated glucose transport in the heart. J. Cell Biochem..

[71] Säll J., Pettersson A.M.L., Björk C., Henriksson E., Wasserstrom S., Linder W., Zhou Y., Hansson O., Andersson D.P., Ekelund M. (2017). Salt-inducible kinase 2 and -3 are downregulated in adipose tissue from obese or insulin-resistant individuals: Implications for insulin signalling and glucose uptake in human adipocytes. Diabetologia.

[72] Säll J., Lindahl M., Fritzen A.M., Fryklund C., Kopietz F., Nyberg E., Warvsten A., Morén B., Foretz M., Kiens B. (2023). Salt-inducible kinases are required for glucose uptake and insulin signaling in human adipocytes. Obesity.

[73] Nixon M., Stewart-Fitzgibbon R., Fu J., Akhmedov D., Rajendran K., Mendoza-Rodriguez M.G., Rivera-Molina Y.A., Gibson M., Berglund E.D., Justice N.J. (2016). Skeletal muscle salt inducible kinase 1 promotes insulin resistance in obesity. Mol. Metab..

[74] Henriksson E., Säll J., Gormand A., Wasserstrom S., Morrice N.A., Fritzen A.M., Foretz M., Campbell D.G., Sakamoto K., Ekelund M. (2015). SIK2 regulates CRTCs, HDAC4 and glucose uptake in adipocytes. J. Cell Sci..

[75] Park J., Yoon Y.-S., Han H.-S., Kim Y.-H., Ogawa Y., Park K.-G., Lee C.-H., Kim S.-T., Koo S.-H. (2014). SIK2 is critical in the regulation of lipid homeostasis and adipogenesis in vivo. Diabetes.

[76] Leitges M. (2002). Knockout of PKC alpha enhances insulin signaling through PI3K. Mol. Endocrinol..

[77] Jensen T.E., Maarbjerg S.J., Rose A.J., Leitges M., Richter E.A. (2009). Knockout of the predominant conventional PKC isoform, PKCalpha, in mouse skeletal muscle does not affect contraction-stimulated glucose uptake. Am. J. Physiol. Endocrinol. Metab..

[78] Deng B., Zhu X., Zhao Y., Zhang D., Pannu A., Chen L., Niu W. (2018). PKC and Rab13 mediate Ca^(2+)^ signal-regulated GLUT4 traffic. Biochem. Biophys. Res. Commun..

[79] Standaert M.L., Bandyopadhyay G., Galloway L., Soto J., Ono Y., Kikkawa U., Farese R.V. (1999). Effects of knockout of the protein kinase C beta gene on glucose transport and glucose homeostasis. Endocrinology.

[80] Kim J.K., Fillmore J.J., Sunshine M.J., Albrecht B., Higashimori T., Kim D.W., Liu Z.-X., Soos T.J., Cline G.E., O’Brien W.R. (2004). PKC-theta knockout mice are protected from fat-induced insulin resistance. J. Clin. Investig..

[81] Peck B., Huot J., Renzi T., Arthur S., Turner M.J., Marino J.S. (2018). Mice lacking PKC-theta in skeletal muscle have reduced intramyocellular lipid accumulation and increased insulin responsiveness in skeletal muscle. Am. J. Physiol. Regul. Integr. Comp. Physiol..

[82] Serra C., Federici M., Buongiorno A., Senni M., Morelli S., Segratella E., Pascuccio M., Tiveron C., Mattei E., Tatangelo L. (2003). Transgenic mice with dominant negative PKC-theta in skeletal muscle: A new model of insulin resistance and obesity. J. Cell Physiol..

[83] Bandyopadhyay G., Standaert M.L., Galloway L., Moscat J., Farese R.V. (1997). Evidence for involvement of protein kinase C (PKC)-zeta and noninvolvement of diacylglycerol-sensitive PKCs in insulin-stimulated glucose transport in L6 myotubes. Endocrinology.

[84] Farese R.V., Sajan M.P., Yang H., Li P., Mastorides S., Gower W.R., Nimal S., Choi C.S., Kim S., Shulman G.I. (2007). Muscle-specific knockout of PKC-lambda impairs glucose transport and induces metabolic and diabetic syndromes. J. Clin. Investig..

[85] Sajan M.P., Bandyopadhyay G., Miura A., Standaert M.L., Nimal S., Longnus S.L., Van Obberghen E., Hainault I., Foufelle F., Kahn R. (2010). AICAR and metformin, but not exercise, increase muscle glucose transport through AMPK-, ERK-, and PDK1-dependent activation of atypical PKC. Am. J. Physiol. Endocrinol. Metab..

[86] Yu H., Fujii N.L., Toyoda T., An D., Farese R.V., Leitges M., Hirshman M.F., Mul J.D., Goodyear L.J. (2015). Contraction stimulates muscle glucose uptake independent of atypical PKC. Physiol. Rep..

[87] Ruby M.A., Riedl I., Massart J., Åhlin M., Zierath J.R. (2017). Protein kinase N2 regulates AMP kinase signaling and insulin responsiveness of glucose metabolism in skeletal muscle. Am. J. Physiol. Endocrinol. Metab..

[88] Rune A., Osler M.E., Fritz T., Zierath J.R. (2009). Regulation of skeletal muscle sucrose, non-fermenting 1/AMP-activated protein kinase-related kinase (SNARK) by metabolic stress and diabetes. Diabetologia.

[89] Fisher J.S., Ju J.-S., Oppelt P.J., Smith J.L., Suzuki A., Esumi H., Tanner C.B., Madsen S.R., Hallowell D.M., Goring D.M.J. (2005). Muscle contractions, AICAR, and insulin cause phosphorylation of an AMPK-related kinase. Am. J. Physiol. Endocrinol. Metab..

[90] Suzuki A., Kusakai G.-I., Kishimoto A., Lu J., Ogura T., Lavin M.F., Esumi H. (2003). Identification of a novel protein kinase mediating Akt survival signaling to the ATM protein. J. Biol. Chem..

[91] Darling N.J., Cohen P. (2021). Nuts and bolts of the salt-inducible kinases (SIKs). Biochem. J..

[92] Verbrugge S.A.J., Alhusen J.A., Kempin S., Pillon N.J., Rozman J., Wackerhage H., Kleinert M. (2022). Genes controlling skeletal muscle glucose uptake and their regulation by endurance and resistance exercise. J. Cell Biochem..

[93] Richter E.A., Vistisen B., Maarbjerg S.J., Sajan M., Farese R.V., Kiens B. (2004). Differential effect of bicycling exercise intensity on activity and phosphorylation of atypical protein kinase C and extracellular signal-regulated protein kinase in skeletal muscle. J. Physiol..

[94] Nielsen J.N., Frøsig C., Sajan M.P., Miura A., Standaert M.L., Graham D.A., Wojtaszewski J.F., Farese R.V., Richter E.A. (2003). Increased atypical PKC activity in endurance-trained human skeletal muscle. Biochem. Biophys. Res. Commun..

[95] Chen H.C., Bandyopadhyay G., Sajan M.P., Kanoh Y., Standaert M., Farese R.V. (2002). Activation of the ERK pathway and atypical protein kinase C isoforms in exercise- and aminoimidazole-4-carboxamide-1-beta-D-riboside (AICAR)-stimulated glucose transport. J. Biol. Chem..

[96] Krisan A.D., Collins D.E., Crain A.M., Kwong C.C., Singh M.K., Bernard J.R., Yaspelkis B.B. (2004). Resistance training enhances components of the insulin signaling cascade in normal and high-fat-fed rodent skeletal muscle. J. Appl. Physiol..

[97] Cao S., Li B., Yi X., Chang B., Zhu B., Lian Z., Zhang Z., Zhao G., Liu H., Zhang H. (2012). Effects of exercise on AMPK signaling and downstream components to PI3K in rat with type 2 diabetes. PLoS ONE.

[98] Kolczynska K., Loza-Valdes A., Hawro I., Sumara G. (2020). Diacylglycerol-evoked activation of PKC and PKD isoforms in regulation of glucose and lipid metabolism: A review. Lipids Health Dis..

[99] Bourbon N.A., Yun J., Kester M. (2000). Ceramide directly activates protein kinase C zeta to regulate a stress-activated protein kinase signaling complex. J. Biol. Chem..

[100] Kajimoto T., Caliman A.D., Tobias I.S., Okada T., Pilo C.A., Van A.A., Andrew McCammon J., Nakamura S.I., Newton A.C. (2019). Activation of atypical protein kinase C by sphingosine 1-phosphate revealed by an aPKC-specific activity reporter. Sci. Signal.

[101] Osada S.I., Mizuno K., Saido T.C., Suzuki K., Kuroki T., Ohno S. (1992). A new member of the protein kinase C family, nPKC theta, predominantly expressed in skeletal muscle. Mol. Cell Biol..

[102] Szendroedi J., Yoshimura T., Phielix E., Koliaki C., Marcucci M., Zhang D., Jelenik T., Müller J., Herder C., Nowotny P. (2014). Role of diacylglycerol activation of PKCtheta in lipid-induced muscle insulin resistance in humans. Proc. Natl. Acad. Sci. USA.

[103] Li Y., Soos T.J., Li X., Wu J., DeGennaro M., Sun X., Littman D.R., Birnbaum M.J., Polakiewicz R.D. (2004). Protein kinase C Theta inhibits insulin signaling by phosphorylating IRS1 at Ser(1101). J. Biol. Chem..

[104] Nomiyama R., Emoto M., Fukuda N., Matsui K., Kondo M., Sakane A., Sasaki T., Tanizawa Y. (2019). Protein kinase C iota facilitates insulin-induced glucose transport by phosphorylation of soluble nSF attachment protein receptor regulator (SNARE) double C2 domain protein b. J. Diabetes Investig..

[105] Cleland P.J.F., Appleby G.J., Rattigan S., Clark M.G. (1989). Exercise-induced translocation of protein kinase C and production of diacylglycerol and phosphatidic acid in rat skeletal muscle in vivo. Relationship to changes in glucose transport. J. Biol. Chem..

[106] Richter E.A., Cleland P.J., Rattigan S., Clark M.G. (1987). Contraction-associated translocation of protein kinase C in rat skeletal muscle. FEBS Lett..

[107] Cleland P.J.F., Abel K.C., Rattigan S., Clark M.G. (1990). Long-term treatment of isolated rat soleus muscle with phorbol ester leads to loss of contraction-induced glucose transport. Biochem. J..

[108] Ihlemann J., Galbo H., Ploug T. (1999). Calphostin C is an inhibitor of contraction, but not insulin-stimulated glucose transport, in skeletal muscle. Acta Physiol. Scand..

[109] Thong F.S., Bilan P.J., Klip A. (2007). The Rab GTPase-activating protein AS160 integrates Akt, protein kinase C, and AMP-activated protein kinase signals regulating GLUT4 traffic. Diabetes.

[110] Watanabe G., Saito Y., Madaule P., Ishizaki T., Fujisawa K., Morii N., Mukai H., Ono Y., Kakizuka A., Narumiya S. (1996). Protein kinase N (PKN) and PKN-related protein rhophilin as targets of small GTPase Rho. Science.

[111] Vincent S., Settleman J. (1997). The PRK2 kinase is a potential effector target of both Rho and Rac GTPases and regulates actin cytoskeletal organization. Mol. Cell Biol..

[112] Flynn P., Mellor H., Casamassima A., Parker P.J. (2000). Rho GTPase control of protein kinase C-related protein kinase activation by 3-phosphoinositide-dependent protein kinase. J. Biol. Chem..

[113] Alessi D.R., James S.R., Downes C.P., Holmes A.B., Gaffney P.R.J., Reese C.B., Cohen P. (1997). Characterization of a 3-phosphoinositide-dependent protein kinase which phosphorylates and activates protein kinase Balpha. Curr. Biol..

[114] Machin P.A., Tsonou E., Hornigold D.C., Welch H.C. (2021). Rho Family GTPases and Rho GEFs in Glucose Homeostasis. Cells.

[115] Moller L.L.V., Klip A., Sylow L. (2019). Rho GTPases-Emerging Regulators of Glucose Homeostasis and Metabolic Health. Cells.

[116] Sylow L., Jensen T.E., Kleinert M., Mouatt J.R., Maarbjerg S.J., Jeppesen J., Prats C., Chiu T.T., Boguslavsky S., Klip A. (2013). Rac1 is a novel regulator of contraction-stimulated glucose uptake in skeletal muscle. Diabetes.

[117] Lundby C., Calbet J.A., Robach P. (2009). The response of human skeletal muscle tissue to hypoxia. Cell Mol. Life Sci..

[118] Azevedo J.L., Carey J.O., Pories W.J., Morris P.G., Dohm G.L. (1995). Hypoxia stimulates glucose transport in insulin-resistant human skeletal muscle. Diabetes.

[119] Cartee G.D., Douen A.G., Ramlal T., Klip A., Holloszy J.O. (1991). Stimulation of glucose transport in skeletal muscle by hypoxia. J. Appl. Physiol..

[120] Mackenzie R., Maxwell N., Castle P., Brickley G., Watt P. (2011). Acute hypoxia and exercise improve insulin sensitivity (S(I) (2*)) in individuals with type 2 diabetes. Diabetes Metab. Res. Rev..

[121] D’Hulst G., Sylow L., Hespel P., Deldicque L. (2015). Acute systemic insulin intolerance does not alter the response of the Akt/GSK-3 pathway to environmental hypoxia in human skeletal muscle. Eur. J. Appl. Physiol..

[122] Wright D.C., Geiger P.C., Holloszy J.O., Han D.-H. (2005). Contraction- and hypoxia-stimulated glucose transport is mediated by a Ca^2+^-dependent mechanism in slow-twitch rat soleus muscle. Am. J. Physiol. Endocrinol. Metab..

[123] Wadley G.D., Lee-Young R.S., Canny B.J., Wasuntarawat C., Chen Z.P., Hargreaves M., Kemp B.E., McConell G.K. (2006). Effect of exercise intensity and hypoxia on skeletal muscle AMPK signaling and substrate metabolism in humans. Am. J. Physiol. Endocrinol. Metab..

[124] Wojtaszewski J.F., Laustsen J.L., Derave W., Richter E.A. (1998). Hypoxia and contractions do not utilize the same signaling mechanism in stimulating skeletal muscle glucose transport. Biochim. Biophys. Acta.

[125] De Groote E., Britto F.A., Balan E., Warnier G., Thissen J.-P., Nielens H., Sylow L., Deldicque L. (2021). Effect of hypoxic exercise on glucose tolerance in healthy and prediabetic adults. Am. J. Physiol. Endocrinol. Metab..

[126] Semenza G.L. (2012). Hypoxia-inducible factors in physiology and medicine. Cell.

[127] Greijer A.E., Van Der Groep P., Kemming D., Shvarts A., Semenza G.L., Meijer G.A., Van De Wiel M.A., Belien J.A., van Diest P.J., van der Wall E. (2005). Up-regulation of gene expression by hypoxia is mediated predominantly by hypoxia-inducible factor 1 (HIF-1). J. Pathol..

[128] Sakagami H., Makino Y., Mizumoto K., Isoe T., Takeda Y., Watanabe J., Fujita Y., Takiyama Y., Abiko A., Haneda M. (2014). Loss of HIF-1alpha impairs GLUT4 translocation and glucose uptake by the skeletal muscle cells. Am. J. Physiol. Endocrinol. Metab..

[129] Görgens S.W., Benninghoff T., Eckardt K., Springer C., Chadt A., Melior A., Wefers J., Cramer A., Jensen J., Birkeland K.I. (2017). Hypoxia in Combination With Muscle Contraction Improves Insulin Action and Glucose Metabolism in Human Skeletal Muscle via the HIF-1alpha Pathway. Diabetes.

[130] Regazzetti C., Peraldi P., Grémeaux T., Najem-Lendom R., Ben-Sahra I., Cormont M., Bost F., Le Marchand-Brustel Y., Tanti J.-F., Giorgetti-Peraldi S. (2009). Hypoxia decreases insulin signaling pathways in adipocytes. Diabetes.

